# Increased cell senescence in human metabolic disorders

**DOI:** 10.1172/JCI169922

**Published:** 2023-06-15

**Authors:** Rosa Spinelli, Ritesh Kumar Baboota, Silvia Gogg, Francesco Beguinot, Matthias Blüher, Annika Nerstedt, Ulf Smith

**Affiliations:** 1 Lundberg Laboratory for Diabetes Research, Department of Molecular and Clinical Medicine, Sahlgrenska Academy, University of Gothenburg, Gothenburg, Sweden.; 2 Department of Translational Medical Sciences, Federico II University of Naples, Naples, Italy.; 3 URT Genomics of Diabetes, Institute of Experimental Endocrinology and Oncology, National Research Council, Naples, Italy.; 4 Evotec International GmbH, Göttingen, Germany.; 5 Helmholtz Institute for Metabolic, Obesity and Vascular Research (HI-MAG), University of Leipzig and University Hospital Leipzig, Leipzig, Germany.

## Abstract

Cell senescence (CS) is at the nexus between aging and associated chronic disorders, and aging increases the burden of CS in all major metabolic tissues. However, CS is also increased in adult obesity, type 2 diabetes (T2D), and nonalcoholic fatty liver disease independent of aging. Senescent tissues are characterized by dysfunctional cells and increased inflammation, and both progenitor cells and mature, fully differentiated and nonproliferating cells are afflicted. Recent studies have shown that hyperinsulinemia and associated insulin resistance (IR) promote CS in both human adipose and liver cells. Similarly, increased CS promotes cellular IR, showing their interdependence. Furthermore, the increased adipose CS in T2D is independent of age, BMI, and degree of hyperinsulinemia, suggesting premature aging. These results suggest that senomorphic/senolytic therapy may become important for treating these common metabolic disorders.

## Introduction

**Figure 1:**
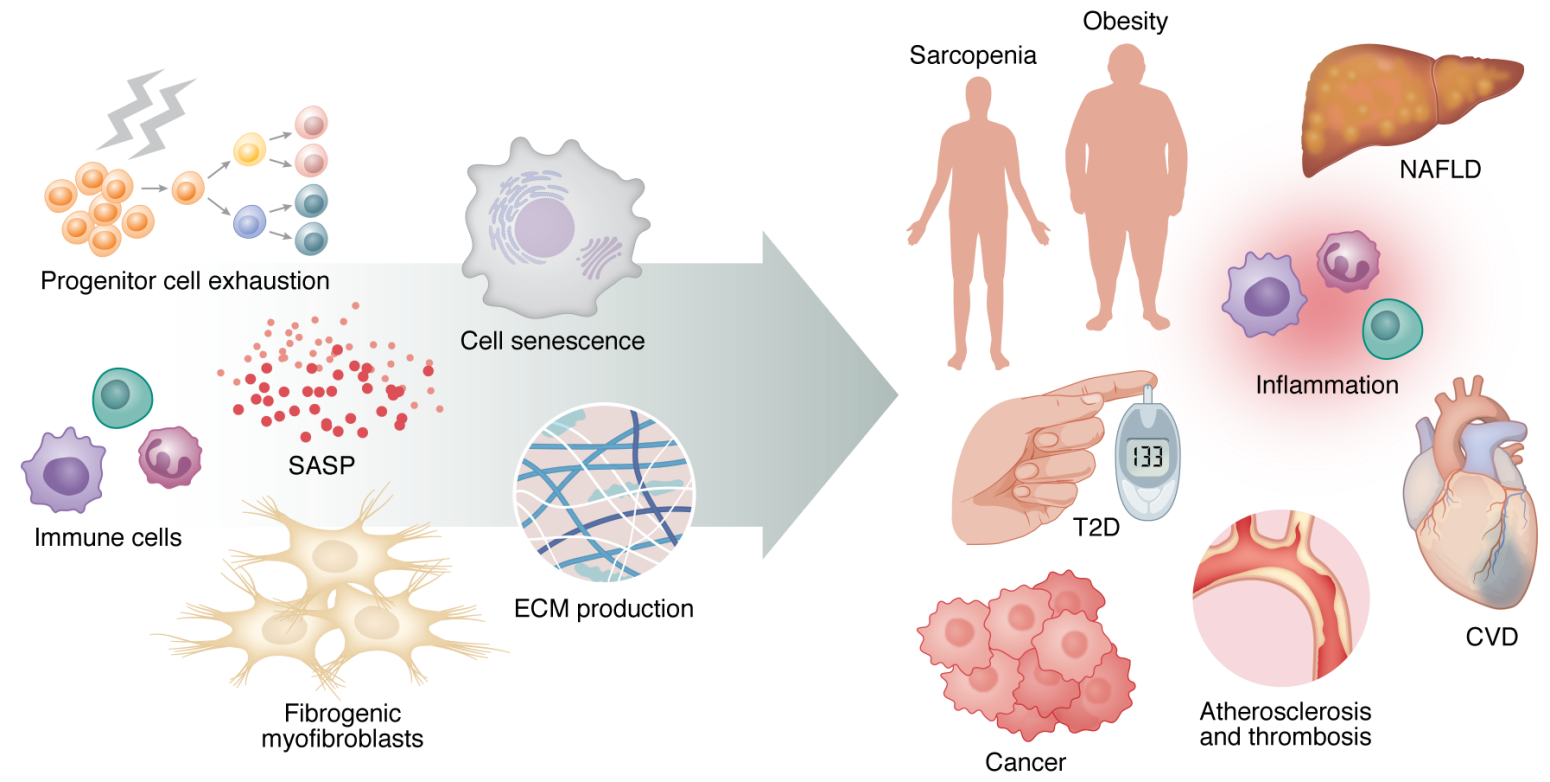
Linking aging to chronic diseases. The hallmarks of aging, including cell senescence, are functionally intertwined and drive the pathophysiology of many chronic disorders, affecting tissues directly involved in disease development. Indeed, senescent cells promote or exacerbate human age-related conditions by inducing chronic inflammation and tissue dysfunction via their SASP. SASP factors are responsible for a number of negative effects, including stem cell dysfunction, immune cell recruitment, fibrogenic myofibroblast induction, and extracellular matrix secretion. The left side of the figure displays the most common cell types and mechanisms that cause tissue damage, all of which contribute to the age-related chronic diseases depicted to the right. The right side of the figure depicts some of the most common chronic diseases associated with aging. CVD, cardiovascular disease.

According to the World Health Organization, the number of people over the age of 80 will triple between 2020 and 2050, reaching around 426 million (1). Aging is a major risk factor for most chronic disorders classified as age-related diseases, including the common metabolic disorders type 2 diabetes (T2D) and nonalcoholic fatty liver disease (NAFLD) and their associated consequences, such as cardiovascular disease and cancer (2). Some age-related diseases have features that resemble premature aging, and many chronic diseases and aging share pathophysiological processes that are fueled by a common set of molecular and cellular mechanisms. These are classified as hallmarks of aging and include genomic instability, telomere attrition, epigenetic alterations, loss of proteostasis, dysregulated nutrient sensing, mitochondrial dysfunction, cell senescence (CS), progenitor cell exhaustion, and altered intercellular communication (1, 3). According to the “geroscience hypothesis,” these hallmarks of aging are functionally intertwined and drive the pathophysiology of many chronic disorders, affecting tissues directly involved in disease development (4) (Figure 1). Based on this, the Unitary Theory of Fundamental Aging Mechanisms proposes that interventions targeting any one hallmark may also target the others, preventing or alleviating multiple age-related diseases as a group rather than one at a time (5). We will focus on CS to illustrate these points. We are aware that CS is unlikely to explain all aging phenotypes. Nonetheless, the senescence response has been linked to a large number of age-related diseases, including metabolic disorders (6). Interventions targeting CS consistently provide therapeutic benefits in preclinical models of obesity, T2D, and NAFLD (7). This is intriguing considering recent findings linking CS to insulin resistance (IR) and associated hyperinsulinemia, both of which are common pathophysiological features of obesity, T2D, and NAFLD (8–13). Thus, CS, which was first described in 1961, has now become a focus for researchers in academia and industry aiming to identify novel agents and strategies to eliminate senescent cells or their effects to be applicable in humans for preventing, delaying, or alleviating multiple metabolic disorders (14, 15).

## Characteristics and markers of senescent cells

CS in adult tissues is a response to damage, limiting proliferation of transformed or aged cells and maintaining homeostasis. Senescent cells are resistant to apoptosis, but they can be cleared by the immune system until it reaches its limit for destruction. According to the threshold theory of senescent cell burden, there is a tipping point beyond which senescent cell number exceeds the immune system’s clearing capacity (15). Senescent cells may then accumulate and interfere with the outcome of physiological and pathological processes, making senescent cell–rich tissues dysfunctional and more vulnerable to additional stressors (16). Indeed, transplanting small numbers of senescent cells causes greater systemic dysfunction in old than in young mice (17). This indicates that a small number of senescent cells is required to promote local tissue dysfunction and systemic deleterious effects and that this minimum number may be related to individual characteristics such as age (18).

CS triggers are numerous and vary based on in vivo context. However, they converge in common pathways that activate the tumor suppressor protein 53 (p53, encoded by *TP53*) to upregulate the cyclin-dependent kinase inhibitor p21 (encoded by *CDKN1A*) and induce signaling networks mediated by p16 (encoded by *CDKN2A*) (14). These cause cell cycle arrest, which is a feature of senescent cells (16). When growth arrest is due to telomere attrition after multiple cell divisions, the senescence is known as replicative senescence. Stress-induced premature senescence is caused by epigenetic changes, DNA damage, bioactive lipids, mitochondrial dysfunction, protein alterations, inflammatory mediators, and other danger signals (6, 19).

Although senescent cells cannot proliferate, they secrete several factors collectively termed the senescence-associated secretory phenotype (SASP). SASP includes a large array of mediators involved in inflammation and tissue damage, including cytokines, chemokines, growth factors, matrix metalloproteinases, and microRNAs. SASP production is controlled at multiple levels (20). The transcriptional level is usually regulated by p38 MAPK, JAK2/STAT3, p53, mTOR, NF-κB, C/EBPβ, and GATA-binding protein 4. Upstream, the cyclic GMP-AMP synthase/stimulator of interferon genes (cGAS/STING) pathway and epigenetic modifications work by regulating chromatin remodeling (21). The composition and destructiveness of SASP can vary depending on the senescent cell type, the nature of the senescence-inducing stimulus, and the surrounding microenvironment. IL-8, IL-6, and TGF-β1 are among the SASP factors by which senescent cells reinforce their own senescence-associated phenotypes and spread senescence to neighboring and distant normal cells (19). Senescent cells with proapoptotic and tissue-destructive SASP can resist self-clearance by activating senescent cell antiapoptotic pathways (SCAPs). Agents that antagonize damaging SASP (senomorphics) or disrupt SCAPs (senolytics), inducing selective apoptosis of senescent cells, show remarkable benefits in preclinical models and promising results in early clinical trials (15).

Senescent cells in vitro are typically enlarged and flattened and have an irregular shape. Their lysosomal compartment is expanded, and this is accompanied by enhanced activity of senescence-associated β-galactosidase (SA-β-gal). Their nuclei show signs of DNA damage, resulting in increased expression of p53, p21, and p16 as well as enhanced phosphorylation of histone variant H2AX (γ-H2AX). Their nuclear integrity is also compromised owing to the loss of lamin B1 (*LMNB1*), and chromatin reorganization caused by epigenetic alterations (19, 22). Although these characteristics are common in senescent cells, they do not always occur at the same time or with the same intensity, making the senescence phenotype extremely dynamic and complex. This heterogeneity highlights the importance of using multiple markers to identify senescent cells.

## Increased senescence in key metabolic human tissues

### Adipose tissue

The adipose tissue (AT) is characterized by increased CS in obesity and T2D, and this was also seen in nondiabetic but insulin-resistant individuals with genetic predisposition for T2D (first-degree relatives) (23). First-degree relatives (FDRs) were not hyperglycemic, so high levels of glucose, which have been shown to induce senescence in various cell types (24), do not appear to be responsible for the elevated AT senescence observed in these subjects. This is consistent with our findings that hyperglycemia has no direct effect on adipocyte senescence induction (25), supporting the idea that multiple factors, not just cell type and senescence inducer, interact to determine senescence outcome (26, 27). The AT is a large and highly active endocrine organ where animal data suggest that CS is initiated in the earliest stages of aging (28). It is composed of a wide variety of cells, including mature adipocytes, which occupy most of the tissue but do not account for more than 20% to 40% of AT-resident cells (29), as well as adipose progenitor cells (APCs; i.e., mesenchymal stem cells and committed preadipocytes), microvascular endothelial cells, and different immune cells. There appears be an annual renewal of around 10% of the adipocytes in humans (30), which means that adipogenesis must be maintained to replace dying cells or recruit new cells needed for increased lipid storage, through differentiation of the progenitor cells (31). If this fails, the remaining adipocytes need to store the excess lipids, leading to inappropriate expansion (hypertrophic obesity), which is well established to be associated with dysfunctional AT, IR, and T2D (32).

**Figure 2:**
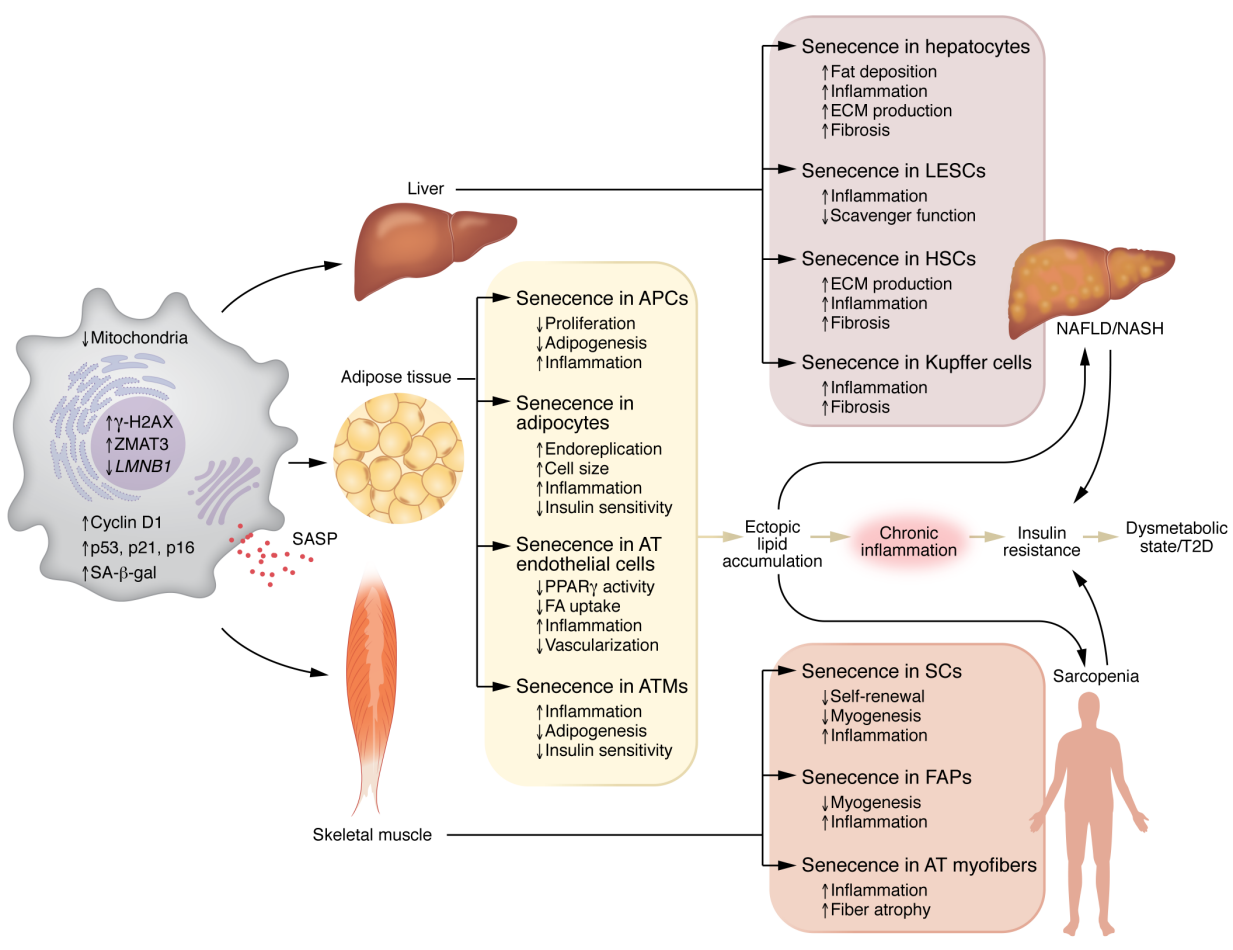
Pathological role of senescent cells in tissue dysfunction and chronic metabolic disease. Cell senescence is a state of cell cycle arrest with senescent cells displaying characteristics such as being enlarged, flattened, and of irregular shape, elevated expression of p53, p21, p16, cyclin D1, and ZMAT3 proteins, increased lysosomal SA-β-gal activity, enhanced γ-H2AX phosphorylation, decreased mitochondrial content, decreased *LMNB1* expression, and SASP acquisition. Increased accumulation of senescent cells in key metabolic tissues (liver, adipose tissue, skeletal muscle) promotes local tissue dysfunction and systemic deleterious effects with IR and the development of common age-related diseases such as T2D, NAFLD/NASH, and sarcopenia. FA, fatty acid.

Excess lipids can be stored in most cells of the body with more or less pronounced negative metabolic and other phenotypic consequences. The appropriately regulated subcutaneous adipose tissue (SAT) is the safest and best site to store excess lipids, while increased storage in the visceral adipose tissue (VAT) is associated with an increased risk of metabolic disease (33). Interestingly, telomere length, as a marker of CS and aging, was found to be shorter in SAT than in VAT, suggesting that SAT is more prone to age-related injuries (34). Increased CS and inflammation have been observed in the AT in both obese mice and humans (23, 35–37). Human SAT from very obese individuals had 7-fold higher SA-β-gal activity than omental AT, and SASP factors were increased and correlated positively with SA-β-gal staining (36). Furthermore, VAT from T2D individuals has increased senescence compared with that from nondiabetic individuals (35). We recently found that SAT biopsies from individuals of similar age but with varying BMI and adipocyte size showed strong correlations between CS and both adipocyte size and degree of IR in vivo, suggesting that adipose CS can promote both hypertrophic expansion and associated IR (23). Furthermore, adipose CS was markedly increased in T2D independent of both age and degree of obesity. We also found increased expression of zinc finger matrin-type 3 (*ZMAT3*), identified as an age-related gene in most human tissues, including SAT (38); and *TP53*, associated with both aging and T2D in two cohorts (39). Taken together, these findings show that AT senescence is increased in obesity, further enhanced in T2D, and associated with IR (Figure 2). A critical question is which cells in the AT become senescent, and the importance of cell-cell crosstalk. Progenitor cells, fully differentiated adipocytes, endothelial cells, macrophages, and likely other immune cells may contribute to CS in AT (40, 41). The number of SAT progenitor cells in individuals with IR, hypertrophic obesity, and T2D did not seem to be reduced, but the adipogenic potential of their APCs was decreased (23). Furthermore, APCs from obese individuals exhibited reduced replicative potential, reduced ability to differentiate, and increased expression of senescence markers and SASP factors compared with APCs from lean individuals (42). This has also been shown in subcutaneous APCs from obese mice (43). For APCs to enter the adipogenic program, p53 must be downregulated, and this is not the case in senescent APCs (23). Importantly, increased APC senescence and reduced ability to downregulate p53 were also commonly seen in nondiabetic FDRs, suggesting that propensity for increased APC senescence is also genetically determined (23). Gustafson et al. also showed that increased senescence in APCs from subjects with obesity and T2D was associated with reduced ability of cells to undergo adipogenesis and normal lipid accumulation (23). The importance of cell-cell crosstalk was also seen in the finding that conditioned medium from senescent cells inhibited adipogenesis in normal cells (23).

Altered DNA methylation is a powerful epigenetic modification that is associated with development of CS (44). In a recent study characterizing the methylome of APCs from FDRs and carefully matched control individuals, we found that FDR cells had several hypomethylated genes related to senescence, with *ZMAT3* as one of the top-ranking genes (45). *ZMAT3* hypomethylation in APCs from FDRs leads to increased cellular levels of ZMAT3 and its close partner p53 (46) as well as increased expression of *CDKN1A* and, consequently, premature senescence (39). Furthermore, ZMAT3 is increased in AT in T2D, and experimentally increased levels of ZMAT3 in human APCs induced senescence and reduced adipogenesis (39). Thus, ZMAT3 is likely an important target for the increased AT senescence in FDRs and T2D.

#### Postmitotic mature adipocytes become senescent and dysfunctional.

Is it possible for postmitotic cells, such as mature adipocytes, to adopt a senescent phenotype even though they have left the cell cycle? A number of studies have shown this to happen in a wide range of postmitotic cells in aging mice, such as in neurons, cardiomyocytes, skeletal muscle myofibers, and osteocytes (47). Several studies in different mouse models have also found that mature adipocytes show markers of increased CS with upregulation of *Trp53* and *Cdkn1a* as well as inflammatory cytokines and increased DNA damage (35, 48, 49). Also, in mice fed a high-fat diet, increased DNA damage and activation of the p53 pathway were seen in mature adipocytes, leading to metabolic and endocrine dysfunction (50). Liu et al. showed that mature adipocytes from mice lacking the negative regulator of p53 triggered a p53-mediated senescent program including SASP factors and impaired adipogenesis (51).

Recent studies confirm that human mature adipocytes also become senescent and dysfunctional. Li et al. found that mature human subcutaneous adipocytes could reenter the cell cycle and synthesize DNA but do not divide, and that this was associated with hyperinsulinemia (12). Adipocytes from obese individuals expressed G_2_-specific cell cycle markers but not markers for the progression through mitosis, which is indicative of an endoreplicative postmitotic cell cycle (12). These data indicate that the cells become arrested in the G_2_ phase, where they exit the cell cycle and undergo senescence. In a recent study, we found that mature human subcutaneous adipocytes in obese individuals adopt a senescent phenotype, and the expression level of CS markers is related to both adipocyte size and degree of IR (25). The association between CS and IR supports a role of the associated hyperinsulinemia, and as we discuss later, chronic hyperinsulinemia induces CS in both mature human adipocytes and human liver cells. However, we also found adipose CS to be markedly more pronounced in adipocytes from individuals with T2D, who did not have higher insulin levels than the included group of matched nondiabetic obese individuals, suggesting factors other than hyperinsulinemia in promoting CS in T2D (25). As discussed above, we have found *ZMAT3* to be increased in adipocytes in T2D, and this may explain the increased CS (25). Additionally, increased senescent adipocytes in T2D were also characterized by increased γ-H2AX staining as a measure of unresolved DNA damage response (DDR), increased SASP factors, and markers of dysfunctional adipocytes, including reduced expression of PPARγ, the glucose transporter GLUT4, and adiponectin, as well as impaired insulin sensitivity measured by phosphorylation of serine/threonine kinase 1 (AKT) on serine 473 (25). To what extent adipocyte senescence is a primary event or a consequence of SASP secretion by senescent cells remains to be shown.

Interestingly, high levels of cyclin D1 (encoded by *CCND1*), an important regulator of cell cycle progression, were detected in obese individuals with hyperinsulinemia (12, 52) as well as adipocytes from individuals with T2D (25). *CCND1*-related transcripts were highly connected to CS, pathways related to cell cycle progression, and DDR, supporting that human mature adipocytes high in cyclin D1 have senesced. Taken together, the increased inflammation and IR in human adipocytes in obesity and T2D may well be promoted by the increased CS.

#### AT endothelial cells.

In the SAT, functional interaction between endothelial cells and adipocytes is fundamental to maintain AT homeostasis. Endothelial cells produce several different proinflammatory factors and pro-atherosclerotic and prothrombotic mediators and express cell adhesion molecules. Senescent endothelial cells secrete higher amounts of growth factors (VEGF, TGF-β), cytokines (IL-6, IL-8, CCL2), adhesion molecules, and matrix proteins. This endothelial cell secretory phenotype contributes to unfavorable vascular remodeling, mediates progressive inflammation (53), and can transmit damaging signals to other vascular cells and tissues (54). Age-related changes in angiogenesis have been considered to result, at least partly, from vascular aging and endothelial CS (55) and are associated with alterations in growth factor signaling and the extracellular matrix (ECM) (56, 57). The growth of AT must be accompanied by a restructure in the vascular network. If the expansion of the AT is excessive, as it is in obesity (which also promotes increased CS in AT cells), the vascularization becomes insufficient, promoting AT dysfunction (32). Furthermore, we have shown that the microvascular endothelial cells in the SAT are specialized cells that, in response to fatty acids, secrete activators of PPARγ (58). The coordinated action of PPARγ activation in endothelial cells and adipocytes enhanced fatty acid uptake and transport and promoted lipid storage in mature adipocytes (58). The ability to respond to increased fatty acids on lipid transport is reduced in senescent AT cells, possibly as a result of decreased endothelial cell function that impairs the coordinated actions of PPARγ activation. Senescent endothelial cells may also lose their ability to secrete endogenous lipid PPARγ activators, which, in turn, would negatively affect preadipocyte differentiation and adipocyte function (59). This crosstalk between human AT endothelial cells and adipocytes needs to be further explored and may be an important reason for the dyslipidemia in obesity and T2D. In animal models, specific deletion of endothelial cell PPARγ prevents fatty acid uptake and leads to systemic hyperlipidemia (60).

#### AT macrophages.

AT macrophages (ATMs) have been linked to obesity and T2D (61). ATM accumulation in SAT is associated with adiposity measures such as BMI and adipocyte size (62). In metabolic disorders, ATMs aggregate in crown-like structures (CLSs) and become a direct source of proinflammatory molecules (62–65). Rabhi et al. revealed that a CD9^+^ subpopulation of ATMs, also found in CLSs, expanded during obesity and became senescent (66). Senescent ATMs secreted profibrotic factors, which also inhibited adipogenesis (66). Interestingly, senolytic treatment of diabetic subjects with chronic kidney disease reduced the abundance of ATMs and CLSs in SAT (67). Recent evidence further shows that accumulation of senescent ATMs in obese subjects is related to BMI, markers of IR, and insulin plasma levels. These data, coupled with the observed ability of senescent macrophages in vitro to propagate inflammation and promote IR in human adipocytes, support a framework in which senescent cells contribute to the development of IR (68).

### Liver

The liver is a complex organ that regulates lipid metabolism, glucose production, and whole-body insulin sensitivity, all of which are perturbed in both T2D and NAFLD (69). NAFLD is a disease of fatty liver but also promotes nonalcoholic steatohepatitis (NASH) with fibrosis, enhancing risk of cirrhosis and hepatocellular carcinoma (HCC). NAFLD is highly prevalent in obesity and in the elderly, closely associated with IR and T2D (70). The negative impact of aging and metabolic disorders on liver health is manifested by progressive impairment of hepatic functions and structural alterations at the cellular level (71, 72). Liver cell abnormalities include volume changes, polyploidy, accumulation of lipofuscin, and a decrease in the number and function of mitochondria. The amount of ECM deposited in the liver increases and contributes to the higher susceptibility for fibrosis (72). All these changes observed during NAFLD development are linked to an increase in hepatic senescence. Indeed, telomere shortening was present in both human and rodent aged liver cells, as well as an increase in p53, p21, and p16 and a loss of *LMNB1* (72). Also, therapeutically targeting CS in the liver in animal models of hepatic disease improved fibrosis, regenerative capacity, and survival (18). However, little is known about CS targeting in human liver disease. Data from rodent and human studies show that senescence develops in several liver cell populations, affecting their functions. In the context of NAFLD, growing evidence in humans indicates that CS appears to occur primarily in hepatocytes, but it can also spread to other cells (73) (Figure 2). Senescent cholangiocytes may also contribute to liver fibrosis (74). However, these cells are primarily involved in cholestatic disorders, and their role in the development of NAFLD is only suggested by studies in mouse models (75). Thus, senescent cholangiocytes are not covered in depth here.

#### Hepatocytes.

Senescent hepatocytes alter liver homeostasis and microenvironment via SASP, which contributes to the development of NAFLD, fibrosis, cirrhosis, and HCC (18, 73). Growing evidence emphasizes their critical role in liver steatosis, likely via mitochondrial dysfunction and impaired lipid metabolism. Indeed, senolytic removal of senescent hepatocytes improved hepatic steatosis in old, obese, and diabetic mice (76). Senescent hepatocytes feature accumulation of damaged mitochondria, polyploidy, and genomic instability as well as loss of proliferative potential and regenerative capacity (73). They also show telomere shortening and dysregulation of p53, p21, and p16 signaling pathways (69). All these abnormalities are accompanied by increased SA-β-gal activity, increased activity of γ-H2AX, and acquisition of a proinflammatory and profibrotic SASP (69, 76). Increased levels of these senescence markers were seen in liver biopsies from NAFLD/NASH patients, and they correlated with disease severity (13, 76). Furthermore, as seen in AT from T2D versus nondiabetic individuals, *ZMAT3* expression is increased together with senescence markers in human liver as the disease progresses from NAFLD to NASH (77), indicating that this gene may also be involved in regulating hepatocyte senescence in human NAFLD/NASH. Baboota et al. recently demonstrated in a large cohort of individuals that increased hepatic senescence is closely associated with liver fat and NALD/NASH grading, as well as whole-body IR and hyperinsulinemia, supporting the role of CS in NAFLD progression and the associated IR (13). Overall, these findings imply that senescent hepatocytes could be therapeutically targeted in NAFLD to reduce IR, steatosis, and fibrosis. In this scenario, evidence that bone morphogenetic protein 4 (BMP4) not only has therapeutic potential in improving whole-body insulin sensitivity and hepatic steatosis, but also has senomorphic anti-senescence effects in human hepatocytes, points to BMP4 and its antagonist gremlin 1 as novel targets to slow hepatocyte senescence, reduce IR, and prevent NAFLD progression (13, 78, 79).

#### Liver sinusoidal endothelial cells.

Liver sinusoidal endothelial cells (LSECs) are endothelial cells that act as a permeable barrier between the blood and hepatocytes by removing soluble macromolecules via the endocytic receptor (80, 81). Although CS has been demonstrated in endothelial cells, there has been no comprehensive study of this process in LSECs. However, aged LSECs have been shown to be in a moderate proinflammatory state, which, along with *Cdkn2a* and γ-H2AX upregulation and downregulation of *Lmnb1*, suggests that these cells senesce during aging (69, 80, 81). Notably, senescent LSECs are not replaced after clearance; thus, their continuous removal may disrupt blood-tissue barriers, resulting in liver fibrosis (80). This evidence supports the concept that delaying senescence is a powerful tool for slowing liver aging. Furthermore, old mice with increased hepatic senescence had impaired LSEC scavenger function, indicating that CS may also contribute to liver sinusoidal dysregulation (80, 81).

#### Hepatic stellate cells.

Hepatic stellate cells (HSCs) are the major fibrogenic hepatic cells. In normal liver, HSCs maintain a nonproliferative, quiescent phenotype. Following injury, HSCs become activated, transdifferentiating to myofibroblasts, which are proliferative, contractile, inflammatory, and characterized by enhanced ECM production (82). Aged HSCs are spontaneously activated, as evidenced by increased proliferation and enhanced expression of fibrosis markers (α-SMA and collagen types I and IV) as well as inflammatory and pro-oxidant factors (82). This causes ECM accumulation, which ultimately contributes to disease aggravation and perpetuation (82). HSCs activated following tissue damage also senesce and are likely cleared, limiting the extent of fibrosis in response to acute injury. However, in response to chronic tissue damage, continuous rounds of hepatocyte death and HSC proliferation allow the production of senescent cells to outpace their clearance, contributing to persistent inflammation and advancing fibrosis (73, 83). Furthermore, the accumulation of senescent hepatocytes and their SASP factors leads to an increase in the number of activated HSCs and, as a result, fibrosis progression with cirrhosis and HCC risk (84, 85). Indeed, even though HSCs may have a dual function in either protecting or promoting HCC (86), it has been demonstrated that nontumoral HSCs in NASH-related HCC expressed higher levels of senescence and SASP markers than nontumoral HSCs in other types of HCCs. Notably, patients with NASH-HCC are significantly older, have higher BMI, and have higher prevalence of T2D than patients with other etiologies of HCC (73), indicating that more research is needed to understand the significance of HSC senescence in relation to aging and metabolic alterations.

#### Kupffer cells.

Kupffer cells (KCs) are resident liver macrophages that serve as key detectors of pathogenic microbial factors, danger signals, and tumor cells moving through the circulation (87). Their number increases with age, and they secrete many proinflammatory cytokines, particularly IL-6. Senescent hepatocytes, through their SASP components, contribute to the progressive increase in KC number. KCs, on the other hand, may cause hepatocyte senescence via IL-6 secretion (87). Overall, these events create a harmful vicious cycle in the aged liver, sustaining NAFLD progression.

### Skeletal muscle

Skeletal muscle (SkM) aids in locomotion and posture and is important for metabolic homeostasis. Its mass and function decline with age owing to a reduction in the number of myofibers and atrophy and weakening of those that remain. These changes cause gait instability and an increased risk of falling and are a major cause of sarcopenia (88). Humans lose 0.7%–0.8% of their muscle mass per year by the age of 60. This loss is associated with impaired muscle function and an increase in intramuscular lipids and surrounding AT (88). Transplanting senescent APCs into young mice reduced grip strength and exercise capacity, while pharmacological and genetic clearance of senescent cells improved SkM performance and physical function during chronological aging (17, 89). Furthermore, combining senolytics with exercise reduced CS and facilitated SkM hypertrophy in older animals, mimicking the exercise response of younger mice (90).

During aging, SkM displays several senescence-initiating factors, including high levels of ROS, telomere shortening, DNA damage, and inflammation. Consistently, SkM from aged humans, monkeys, and mice expresses increased levels of senescence markers, including p53 and p21 (7). SkM is composed of postmitotic multinucleated cells (myofibers) and a mix of mitotically competent mononuclear cells. However, the susceptibility of these cell populations to senescence with age is unclear. Moiseeva et al. revealed that the SkM cell types most enriched for senescence markers are monocytes, macrophages, myeloid cells, fibro-adipogenic progenitors, stem cells (called satellite cells), and their progeny (91). However, the bulk of evidence for core properties of the senescence program activated in the context of SkM aging and disease is related to fibro-adipogenic progenitors, satellite cells, and myofibers (7) (Figure 2).

#### Satellite cells.

Satellite cells (SCs) are normally quiescent but can enter the cell cycle after injury and proliferate, forming new myofibers and replenishing the stem cell pool (92). The regenerative capacity of SCs declines with age. This is due to a continued decrease in the number of SCs and an impairment in their activation/proliferation in response to damage-inducing stimuli in both mice and humans as they age, which appears to be caused in part by cell-extrinsic factors (7). These include SASP factors, as the lower ability of aged SCs to remain quiescent is associated with increased expression of senescence markers. Indeed, reducing senescence and inflammation with senolytics improved SkM wasting and strength in vivo as well as the proliferative capability of cultured SCs (89).

#### Fibro-adipogenic progenitors.

Fibro-adipogenic progenitors (FAPs) are mesenchymal progenitor cells with fibrogenic and adipogenic potential. They were required for myogenic commitment and SkM repair following injury or exercise (7). Interestingly, young, but not old, FAP transplantation restored the myogenic potential of aged SCs. This evidence demonstrates that FAPs change with age, emphasizing the importance of FAP-derived signals in SkM health (93). Data from progeroid mice show that FAPs become senescent and impair SC function in a paracrine manner. Consistently, senolytic depletion of senescent FAPs in the same mouse model restored the pool of SCs and their function (94). Single-cell and bulk RNA sequencing, as well as complementary imaging methods on SkM from old mice, identified a specific FAP subpopulation characterized by upregulation of multiple senescence-related genes (*Cdkn1a*, *Cdkn2a*), SASP factors (IL-6, TNF-α, PAI-1, MMP-3, MMP-9, TGF-β1), SASP effector pathways, DNA damage markers (γ-H2AX), and chromatin reorganization (*Lmnb1* loss), definitively proving that a subset of FAPs become senescent with age (94).

#### Myofibers.

Increased senescence markers were found in myofiber cross sections of aged SkM in mice, and further investigation revealed correlations between these markers and fiber atrophy parameters (7). Senescent cell transplantation in immunodeficient NOD/SCID mice resulted in induction of senescence in adjacent myofibers, suggesting a substantial impact of the bystander effect for accumulation of senescent myofibers in SkM aging (7). Consistent with this finding, Zhang et al. recently demonstrated that an age-dependent increase in *Cdkn1a* and hyperactivation of senescence-associated pathways, including the p53 signaling and cytokine-related pathways, occur in a subpopulation of terminally differentiated SkM myofibers from old mice (95). Treatment of progeroid mice with senolytics reversed the molecular features of senescence not only in myofibers but also in FAPs, improving SkM health parameters (94). Increased senescence markers were also discovered in the SkM of the elderly. Human studies, like mouse data, show a significant inverse correlation between *CDKN2A* and *CDKN1A* expression and SkM quality and function (95). Overall, these findings show that CS is a conserved mechanism of SkM aging in mice and humans, and that it plays a pathogenic role in age-related SkM dysfunction.

## IR/hyperinsulinemia promotes CS in human cells

**Figure 3:**
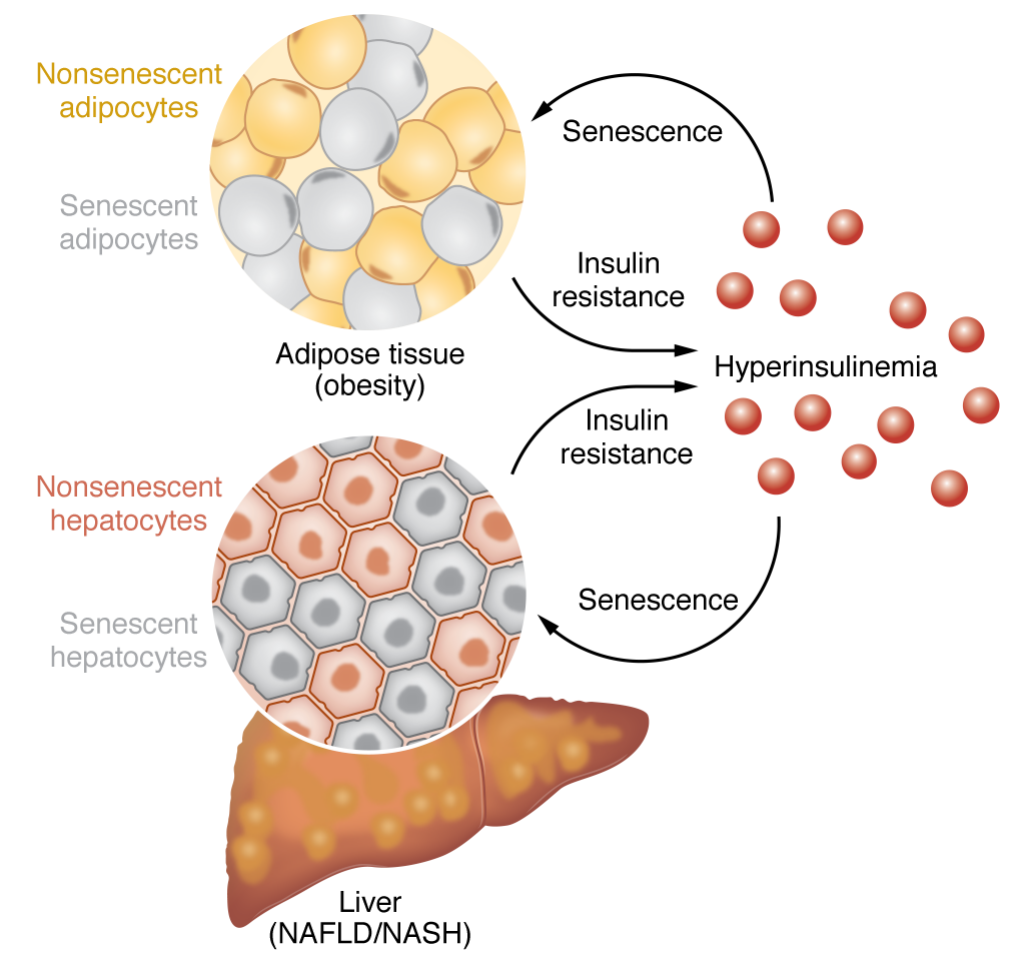
Cell senescence as likely mediator of the bidirectional relationship between IR and hyperinsulinemia. Senescent cells accumulate in multiple tissues in obesity, T2D, and NAFLD/NASH, including AT and the liver. Increased CS in those tissues may cause IR, which, in turn, leads to hyperinsulinemia. Hyperinsulinemia, on the other hand, may induce senescence in both adipocytes and liver cells, contributing to an increase in CS burden in the AT and liver. Thus, CS may enable and fuel a vicious cycle between IR and hyperinsulinemia, which plays a considerable role in the development of several metabolic diseases and their consequences.

IR and hyperinsulinemia are major features of several metabolic disorders, including obesity, T2D, NAFLD, and their associated comorbidities. Although IR and hyperinsulinemia are closely interrelated, their temporal sequence is still unclear. IR is generally thought of as the primary cause of hyperinsulinemia. However, both clinical and experimental studies have shown that hyperinsulinemia may also precede and promote IR (96, 97). The emerging consensus is that IR and hyperinsulinemia have a bidirectional relationship, which may contribute to the development and progression of metabolic disorders (12, 13, 25). Recent studies have shown that the CS increase seen in obesity, T2D, and NAFLD in metabolic tissues was significantly associated with whole-body IR and hyperinsulinemia in humans (12, 13, 25). The role of insulin was further supported by recent experimental evidence in vitro showing that hyperinsulinemia induced early senescence in both human adipose and liver cells (12, 98, 99). Studies in both humans and mice suggested that this could also happen in vivo (12, 98, 100). Senescent cells have also been directly linked to the development of IR, as demonstrated with the use of senolytics in several preclinical models of aging and metabolic disease (15, 101). This suggests that the observed bidirectional relationship between IR and hyperinsulinemia may be explained by CS, pointing to IR and hyperinsulinemia as mutually reinforcing components of a vicious cycle fueled by elevated CS (Figure 3). Some of the seminal studies that support this model are discussed below. However, a note of caution is important, as much of our understanding of the contribution of senescent cells to IR comes from animal models for human conditions, and this needs to be documented also in humans.

Mounting evidence demonstrated a causal role of senescent cells within AT in the development of IR. For instance, Gustafson et al. showed that mature adipocytes from obese individuals displayed increased senescence, and this was considerably further enhanced in equally obese and aged individuals with T2D (25). Notably, the degree of adipocyte senescence was positively correlated with whole-body IR, supporting the concept that CS promotes IR (25). Mechanistically, p53 upregulation in adipocytes promotes IR, whereas its inhibition ameliorated senescence-like changes and improved IR (35). Furthermore, p53 inhibition using pifithrin-a or adipose DNA damage reduction by *N*-acetylcysteine or metformin ameliorated adipocyte senescence and reduced metabolic abnormalities (48).

The importance of hyperinsulinemia in the pathogenesis of CS has been gaining momentum recently. Li et al. demonstrated that continuous exposure of mature adipocytes from obese individuals to hyperinsulinemia caused cyclin D1–induced G_1_/S cell cycle progression, but not mitosis, leading to endoreplication and induction of a senescent cell program (12). Recently, Baboota et al. reported a strong association between increase in fasting insulin levels, as well as in degree of IR, and hepatic CS in obese NAFLD/NASH patients (13). In a subsequent study, using an in vitro model, the authors revealed that chronic hyperinsulinemia initiated a senescence cell program in human hepatocytes via enhanced *CCND1* expression and the p53/p21 signaling pathway (98). Additionally, a recent study with two independent NAFLD/NASH cohorts showed that plasma insulin levels were strongly associated with markers of hepatic CS (100). Taken together, these studies support the importance of chronic hyperinsulinemia and IR in promoting human adipose and liver CS, and that this effect is independent of aging. However, it should be emphasized that the markedly increased CS in the AT cells in T2D is unlikely to be a consequence of hyperinsulinemia alone, as insulin levels were not higher in the matched T2D individuals compared with the obese nondiabetic individuals (25, 84). The mechanism for this remains to be identified.

A recent study using LIRKO mice (a model of pure IR in hepatocytes associated with hyperinsulinemia and a consequence of insulin receptor deletion in the liver) revealed that senescence markers were strongly reduced in liver cells lacking insulin receptors, whereas an increase in senescence was observed in the AT of the same mice (98). These findings support the concept that insulin-dependent signaling pathways are essential for the induction of senescence by hyperinsulinemia. Furthermore, the shared mechanisms between development of IR, hyperinsulinemia, and CS suggest that these are not mutually exclusive concepts and most likely act in parallel.

## Conclusions and future perspectives

CS is generally driven by aging and is strongly associated with age-related disorders. It promotes the common age-associated phenotypes of reduced number of functional cells and size of tissues/organs, increased fibrosis, inflammation, and accumulation of cells with genetic defects. However, other (epi)genetic disorders such as T2D, obesity, and NAFLD/NASH are also characterized by increased CS in metabolic cells.

CS is induced by several known factors, but it was recently shown that hyperinsulinemia, commonly associated with IR, also induces CS in human metabolic cells. Similarly, increased CS induces IR, showing the bidirectional pathway between CS and IR.

Despite a low percentage of senescent cells in tissues, their SASP can further spread senescence locally or distally, leading to a vicious cycle of senescent cell formation. Senotherapeutic strategies, mainly senolytic, have shown great potential in alleviating metabolic and other complications in preclinical animal models. However, we need well-controlled clinical studies to really understand their potential in metabolic disorders such as T2D. Furthermore, it is crucial to recognize the heterogeneity of senescence among cell types to improve disease-specific targeting of CS. Advanced technologies are expanding our understanding of CS, enabling us to develop rational design strategies to target and eliminate detrimental senescent cell subpopulations, which may extend average human healthspan.

## Version 1 06/15/2023

Electronic publication
